# Functional polymorphisms of the *TET1* gene increase the risk of neuroblastoma in Chinese children

**DOI:** 10.1111/jcmm.17820

**Published:** 2023-06-22

**Authors:** Jiaming Chang, Lei Lin, Chunlei Zhou, Xinxin Zhang, Tianyou Yang, Haiyan Wu, Yan Zou, Jing He

**Affiliations:** ^1^ Department of Pediatric Surgery, Guangzhou Institute of Pediatrics, Guangdong Provincial Key Laboratory of Research in Structural Birth Defect Disease, Guangzhou Women and Children's Medical Center Guangzhou Medical University, Guangdong Provincial Clinical Research Center for Child Health Guangzhou China; ^2^ Department of Pathology Children's Hospital of Nanjing Medical University Nanjing China

**Keywords:** m5C modification, neuroblastoma, polymorphism, susceptibility, *TET1*

## Abstract

Common genetic mutations are absent in neuroblastoma, one of the most common childhood tumours. As a demethylase of 5‐methylcytosine (m5C) modification, TET1 plays an important role in tumourigenesis and differentiation. However, the association between *TET1* gene polymorphisms and susceptibility to neuroblastoma has not been reported. Three *TET1* gene polymorphisms (rs16925541 A > G, rs3998860 G > A and rs12781492 A > C) in 402 Chinese patients with neuroblastoma and 473 cancer‐free controls were assessed using TaqMan. Multivariate logistic regression analysis was used to evaluate the association between *TET1* gene polymorphisms and susceptibility to neuroblastoma. The GTEx database was used to analyse the impact of these polymorphisms on peripheral gene expression. The relationship between gene expression and prognosis was analysed using Kaplan–Meier analysis with the R2 platform. We found that both rs3998860 G > A and rs12781492 A > C were significantly associated with increased neuroblastoma risk. Stratified analysis further showed that rs3998860 G > A and rs12781492 A > C significantly increased neuroblastoma risk in certain subgroups. In the combined risk genotype model, 1–3 risk genotypes significantly increased risk of neuroblastoma compared with the 0 risk genotype. rs3998860 G > A and rs12781492 A > C were significantly associated with increased STOX1 mRNA expression in adrenal and whole blood, and high expression of STOX1 mRNA in adrenal and whole blood was significantly associated with worse prognosis. In summary, *TET1* gene polymorphisms are significantly associated with increased neuroblastoma risk; further research is required for the potential mechanism and therapeutic prospects in neuroblastoma.

## 1 INTRODUCTION

Neuroblastoma is an embryonic tumour of the sympathetic nervous system, but its origin is unclear.^1^ Currently, it is generally believed that it originates from incomplete precursors of neural crest tissue.^2^, ^3^, ^4^ Neuroblastoma is the most common solid tumour and the second most common extracranial tumour in children.^5^, ^6^ In developed countries, the incidence of neuroblastoma in children aged 0–14 years is approximately 10.1–15.0 per 1 million.^7^ In China, the incidence of neuroblastoma is 7.7 cases per 1 million children.^8^ Neuroblastoma exhibits a high degree of heterogeneity, from biological characteristics to clinical processes.^1^ Some neuroblastomas can spontaneously subside.^1^ Even after a series of intensive treatments, such as surgery, immunotherapy and radiation therapy, high‐risk neuroblastomas still have a relatively poor prognosis and high risk of recurrence.^9^, ^10^, ^11^ Patients with high‐risk neuroblastoma will also face serious psychological problems such as anxiety.^12^ Identifying biological characteristics associated with high‐risk neuroblastoma is a research focus. In addition to *MYCN* amplification, some important other genetic changes have been found in neuroblastomas, such as *ATRX*,^13^ *TERT*,^14^ *ALK* ^15^ and *RAS* ^16^ mutations. However, data thus far are insufficient to explain the genetic variations associated with neuroblastoma risk.

As the most common genetic variation in DNA, single‐nucleotide polymorphisms (SNPs) are closely related to tumour susceptibility, prognosis and immunity.^17^, ^18^, ^19^ Different tumour types have different associated SNPs.^20^ Genome‐wide association studies (GWASs) have enabled large‐scale exploration of SNPs truly related to tumours in the entire genome. With the application of GWAS to neuroblastoma, many SNPs have been proven to be related to its susceptibility.^21^, ^22^, ^23^, ^24^ John et al.^24^ have shown that rs2168101 G > T located in the *LMO1* gene super‐enhancer is closely related to genetic susceptibility to neuroblastoma; the rs2168101 T allele is associated with reduced *LMO1* expression and tumour suppression in primary neuroblastoma tumours. However, the genetic variations associated with neuroblastoma susceptibility in different populations are yet to be elucidated. Our previous studies have shown that the BER pathway and *ERCC1*, *XPF*, *NRAS* and *ALKBH5* gene polymorphisms affect neuroblastoma risk in Chinese children.^16^, ^25^, ^26^, ^27^ However, the genetic polymorphisms associated with the risk of neuroblastoma in Chinese children are not fully understood.

Epigenetic modifications commonly found in genomes and transcriptomes have been shown to play important biological roles in growth and development, aging and various diseases.^28^, ^29^, ^30^, ^31^ In recent years, RNA modifications have been shown to play an important role in cancer.^28^, ^32^ Common RNA modifications include N6‐methyladenosine (m6A), N1‐methyladenosine (m1A), 5‐methylcytosine (m5C), 5‐hydroxymethylcytosine (hm5C), pseudouridine (Ψ) and N7‐methylguanosine (m7G). As the most common DNA modification and important RNA modification, the role of m5C modification in cancer cannot be ignored.^29^, ^33^, ^34^, ^35^ As m5C demethylases, the ten‐eleven translocation (TET) family of enzymes oxidize m5C in RNA to form hm5C.^36^ TET further oxidizes 5hmC to 5fC (5‐formylcytosine) and 5caC (5‐carboxycytidine) in DNA^37^; thymidine DNA glycosylase recognizes 5fC and 5caC and converts them to unmethylated cytosine.^38^, ^39^, ^40^ A member of the TET (ten‐eleven translocation) family, TET1 (tet methylcytosine dioxygenase 1) has been shown to play an important biological role in cancer, including neuroblastoma, in recent years.^41^, ^42^, ^43^, ^44^ Fragile X mental retardation protein (FMRP) promotes demethylation of m5C by interacting with TET1 and induces transcription‐coupled homologous recombination, which is an important mechanism for mRNA repair and cell survival in cancer.^41^ The oncoprotein YAP induces expression of TET1, which promotes transcriptional activation and induces tumourigenesis through epigenetics, such as DNA demethylation caused by its interaction with TEAD.^42^ Gao et al.^44^ found that TET1 correlated negatively with neuronal differentiation in neuroblastoma cells. TET1 mediates negative regulation of neuronal differentiation by srGAP3 through non‐catalytic action.^44^ The *TET1* gene is repeatedly mutated in lung cancer, gastrointestinal cancer, skin cancer and urinary system cancer.^45^ In cancer patients treated with immune checkpoint inhibitors (ICIs), the *TET1* mutation is associated with better therapeutic efficacy.^45^ This suggests a potential therapeutic target for the *TET1* gene. However, the important role of *TET1* gene mutations in neuroblastoma needs to be further explored. Currently, there is no research indicating that *TET1* gene polymorphisms are associated with susceptibility to neuroblastoma.

Based on the importance of the *TET1* gene in neuroblastoma, we hypothesize that *TET1* gene polymorphisms have an impact on risk of neuroblastoma. To test our hypothesis, we conducted a case–control study in Jiangsu, China, to explore whether *TET1* gene polymorphisms are associated with susceptibility to neuroblastoma.

## 2 MATERIALS AND METHODS

### 2.1 Study subjects

This case–control study included 402 children with neuroblastoma and 473 noncancer children (Table S1) recruited from the Children's Hospital of Nanjing Medical University in Jiangsu Province.^46^ Epidemiological data were collected using structured questionnaires. The inclusion criterion for patients was neuroblastoma confirmed by biopsy or histology. The control group subjects were matched based on the expected population characteristic (sex, age and race) distribution of the case group and recruited simultaneously with the case group. For our study, all subjects signed an informed consent form. This study was approved by the Institutional Review Committee of Children's Hospital of Nanjing Medical University (Approval No.: 202112141–1).

### 2.2 Polymorphisms selection and genotyping

We first selected potential functional SNPs located in the 5′ flanking region, 5′ untranslated region (UTR), exon, intron and 3′ UTR regions of the *TET1* gene from the dbSNP database and SNPinfo website. The secondary allele frequency of all SNPs in the Han Chinese population reported in 1000 Genomes is >5%. Linkage disequilibrium (LD) between the selected SNPs is less than 0.8. The final SNPs selected were rs16925541, rs3998860 and rs12781492. Both rs16925541 and rs3998860 are missense variants located in the coding region of the *TET1* gene. The variant rs12781492 is located in the 3′ UTR of the *TET1* gene and is predicted to bind to miRNA. We used TIANamp Blood DNA Kit (TianGen Biotech Co. Ltd.) to extract genomic DNA from tissue or blood samples from all subjects. The concentration and purity of the extracted DNA were measured using a UV spectrophotometer (Nano Drop Technologies, Inc.). The DNA sample was diluted and transferred to a 96‐well plate. For all samples, TaqMan® SNP Genotyping Assays (Applied Biosystems) were used for genotyping. We randomly selected 10% of the samples from the case and the control groups for repeated genotyping, and the results were 100% consistent.

### 2.3 Statistical analysis

Differences in genotype frequency distribution and demographic characteristics between the case group and the control group were analysed using a bilateral chi‐square test. The goodness of fit χ^2^ test was used for Hardy–Weinberg equilibrium (HWE). To explore the association between *TET1* gene polymorphisms and susceptibility to neuroblastoma, we used multiple logistic regression analysis to analyse the odds ratio (OR) and 95% confidence interval (CI) of *TET1* gene polymorphisms in the case group and the control group. The OR calculates the crude OR and adjusted OR before and after adjustment for age and sex, respectively. Subsequently, further stratification analysis was conducted based on age, sex, tumour location and clinical stage for significant SNPs. The above statistical analyses were performed using SAS V9.4 (SAS Institute). Gene expression quantitative trait loci (eQTLs) were analysed through the Genotype‐Tissue Expression (GTEx) official website (https://www.gtexportal.org/home/). Analysis of the relationship between gene expression and prognosis of neuroblastoma cases derived from the GSE62564 dataset using Kaplan–Meier analysis through R2: Genomics Analysis and Visualization Platform (http://r2.amc.nl). All statistics were conducted using a two‐sided test with a significance level of 0.05.

## 3 RESULTS

### 3.1 Associations of *TET1* gene polymorphisms with neuroblastoma susceptibility

We successfully obtained the genotypes of the three SNPs in 400 cases and 473 controls. As shown in Table 1, all SNPs in the control group conformed to HWE (*p* > 0.05). After adjusting for age and sex, we found that subjects with the rs3998860 AA genotype had a 1.5‐fold increased neuroblastoma risk compared with those with the GG genotype (AA vs. GG: adjusted OR = 2.51, 95% CI = 1.27–4.95, *p* = 0.008). We also found that the recessive model of the SNP rs3998860 was significantly associated with increased neuroblastoma risk (AA vs. GG/GA: adjusted OR = 2.69, 95% CI = 1.37–5.27, *p* = 0.004). Compared with the rs12781492 AA genotype, the rs12781492 CC genotype was significantly associated with increased neuroblastoma risk (CC vs. AA: adjusted OR = 2.04, 95% CI = 1.01–4.10, *p* = 0.046). Compared to the rs12781492 AA/AC genotypes, the CC genotype also significantly increased neuroblastoma risk (CC vs. AA/AC: adjusted OR = 2.17, 95% CI = 1.08–4.34, *p* = 0.029). Based on the above statistical analysis results, we defined rs16925541 GG, rs3998860 AA and rs12781492 CC as risk genotypes to further analyse the impact of combined genotypes on susceptibility to neuroblastoma. The results showed that the combination of 1–3 risk genotypes significantly increased neuroblastoma susceptibility compared to the 0 risk genotype (1–3 vs. 0: adjusted OR = 3.01, 95% CI = 1.67–5.40, *p* = 0.0002).

**TABLE 1:** Association of *TET1* gene polymorphisms with neuroblastoma risk in children from Jiangsu province.

Genotype	Cases (*n* = 400)	Controls (*n* = 473)	*p* ^a^	Crude OR (95% CI)	*p*	Adjusted OR (95% CI)^b^	*p* ^b^
rs16925541 A > G (HWE = 0.142)							
AA	308 (77.00)	365 (77.17)		1.00		1.00	
AG	77 (19.25)	97 (20.51)		0.94 (0.67–1.32)	0.722	0.94 (0.67–1.32)	0.722
GG	15 (3.75)	11 (2.33)		1.62 (0.73–3.57)	0.235	1.63 (0.73–3.60)	0.231
Additive			0.641	1.07 (0.82–1.39)	0.640	1.07 (0.82–1.39)	0.637
Dominant	92 (23.00)	108 (22.83)	0.953	1.01 (0.74–1.39)	0.953	1.01 (0.74–1.39)	0.952
AA/AG	385 (96.25)	462 (97.67)		1.00		1.00	
GG	15 (3.75)	11 (2.33)	0.217	1.64 (0.74–3.61)	0.221	1.65 (0.75–3.64)	0.217
rs3998860 G > A (HWE = 0.582)							
GG	276 (69.00)	319 (67.44)		1.00		1.00	
GA	96 (24.00)	141 (29.81)		0.79 (0.58–1.07)	0.124	0.79 (0.58–1.07)	0.128
AA	28 (7.00)	13 (2.75)		**2.49 (1.26–4.90)**	**0.008**	**2.51 (1.27–4.95)**	**0.008**
Additive			0.488	1.09 (0.86–1.37)	0.487	1.09 (0.86–1.38)	0.478
Dominant	124 (31.00)	154 (32.56)	0.623	0.93 (0.70–1.24)	0.623	0.93 (0.70–1.24)	0.628
GG/GA	372 (93.00)	460 (97.25)		1.00		1.00	
AA	28 (7.00)	13 (2.75)	0.003	**2.66 (1.36–5.21)**	**0.004**	**2.69 (1.37–5.27)**	**0.004**
rs12781492 A > C (HWE = 0.279)							
AA	303 (75.75)	348 (73.57)		1.00		1.00	
AC	74 (18.50)	112 (23.68)		0.76 (0.55–1.06)	0.103	0.76 (0.54–1.06)	0.103
CC	23 (5.75)	13 (2.75)		**2.03 (1.01–4.08)**	**0.046**	**2.04 (1.01–4.10)**	**0.046**
Additive			0.822	1.03 (0.80–1.32)	0.822	1.03 (0.80–1.32)	0.819
Dominant	97 (24.25)	125 (26.43)	0.462	0.89 (0.66–1.21)	0.462	0.89 (0.66–1.21)	0.462
AA/AC	377 (94.25)	460 (97.25)		1.00		1.00	
CC	23 (5.75)	13 (2.75)	0.026	**2.16 (1.08–4.32)**	**0.030**	**2.17 (1.08–4.34)**	**0.029**
Combine risk genotypes^c^							
0	360 (90.00)	456 (96.41)		1.00		1.00	
1–3	40 (10.00)	17 (3.59)	0.0001	**2.98 (1.66–5.34)**	**0.0002**	**3.01 (1.67–5.40)**	**0.0002**

*Note*: Values are in bold if the *p* values are less than 0.05 or the 95% CIs excluded 1.

Abbreviations: CI, confidence interval; HWE, Hardy–Weinberg equilibrium; OR, odds ratio.

^a^ χ^2^ test for genotype distributions between neuroblastoma patients and cancer‐free controls.

^b^ Adjusted for age and sex.

^c^ Risk genotypes were carriers with rs16925541 GG, rs3998860 AA and rs12781492 CC genotypes.

### 3.2 Stratification analysis of significant SNPs

We further analysed the impact of *TET1* gene polymorphisms on susceptibility to neuroblastoma by stratified analysis of age, sex, site of origin and clinical stage (Table 2). Our results suggested that the rs3998860 AA genotype significantly increases risk of neuroblastoma in children compared to the GG/GA genotype in the >18 month subgroup (AA vs. GG/GA: adjusted OR = 3.07, 95% CI = 1.16–8.12, *p* = 0.024). Similarly, the rs3998860 AA genotype significantly increased risk of neuroblastoma in male children in sex stratification (AA vs. GG/GA: adjusted OR = 3.57, 95% CI = 1.26–10.14, *p* = 0.017), the retroperitoneal subgroup of sites in origin stratification (AA vs. GG/GA: adjusted OR = 4.03, 95% CI = 1.91–8.51, *p* = 0.0003), and I + II + 4 s (AA vs. GG/GA: adjusted OR = 3.00, 95% CI = 1.35–6.64, *p* = 0.007) and III + IV (AA vs. GG/GA: adjusted OR = 2.69, 95% CI = 1.17–6.17, *p* = 0.019) subgroups in clinical stage stratification. The recessive model of rs12781492 was significantly associated with increased neuroblastoma risk in the retroperitoneal subgroup (CC vs. AA/AC: adjusted OR = 2.75, 95% CI = 1.23–6.15, *p* = 0.014) and the III + IV subgroup (CC vs. AA/AC: adjusted OR = 2.64, 95% CI = 1.15–6.04, *p* = 0.022). In all subgroups stratified by age (≤18 months: adjusted OR = 3.22, 95% CI = 1.32–7.90, *p* = 0.011; >18 months: adjusted OR = 2.67, 95% CI = 1.23–5.80, *p* = 0.013), sex (females: adjusted OR = 2.97, 95% CI = 1.33–6.67, *p* = 0.008; males: adjusted OR = 3.05, 95% CI = 1.30–7.14, *p* = 0.010) and clinical stage (I + II + 4 s: adjusted OR = 2.98, 95% CI = 1.48–6.01, *p* = 0.002; III + IV: adjusted OR = 3.03, 95% CI = 1.49–6.19, *p* = 0.002), a combination of 1–3 risk genotypes was significantly associated with increased neuroblastoma risk compared to the 0 risk genotype. Combination of 1–3 risk genotypes significantly increased neuroblastoma risk in the retroperitoneal subgroup compared to the 0 risk genotype (1–3 vs. 0: adjusted OR = 4.54, 95% CI = 2.37–8.70, *p* < 0.0001).

**TABLE 2:** Stratification analysis for the association between *TET1* risk genotypes with neuroblastoma susceptibility in Jiangsu children.

Variables	rs3998860 (cases/controls)		Adjusted OR^a^	*p* ^a^	rs12781492 (cases/controls)		Adjusted OR^a^	*p* ^a^	Risk genotypes (cases/controls)		Adjusted OR^a^	*p* ^a^
	GG/GA	AA	(95% CI)		AA/AC	CC	(95% CI)		0	1–3	(95% CI)	
Age, month												
≤18	124/132	14/7	2.15 (0.84–5.49)	0.111	127/134	10/5	2.11 (0.70–6.34)	0.184	117/132	20/7	**3.22 (1.32–7.90)**	**0.011**
>18	249/328	14/6	**3.07 (1.16–8.12)**	**0.024**	250/326	13/8	2.12 (0.86–5.19)	0.101	243/324	20/10	**2.67 (1.23–5.80)**	**0.013**
Sex												
Females	177/217	14/8	2.15 (0.88–5.24)	0.093	178/219	13/6	2.67 (0.99–7.16)	0.052	170/216	21/9	**2.97 (1.33–6.67)**	**0.008**
Males	195/243	14/5	**3.57 (1.26–10.14)**	**0.017**	199/241	10/7	1.75 (0.65–4.69)	0.267	190/240	19/8	**3.05 (1.30–7.14)**	**0.010**
Sites of origin												
Adrenal gland	89/460	4/13	1.66 (0.53–5.23)	0.389	90/460	3/13	1.19 (0.33–4.27)	0.789	88/456	5/17	1.55 (0.56–4.33)	0.401
Retroperitoneal	149/460	17/13	**4.03 (1.91–8.51)**	**0.0003**	154/460	12/13	**2.75 (1.23–6.15)**	**0.014**	142/456	24/17	**4.54 (2.37–8.70)**	**<0.0001**
Mediastinum	113/460	6/13	1.91 (0.71–5.17)	0.202	112/460	7/13	2.23 (0.87–5.72)	0.096	110/456	9/17	2.21 (0.96–5.11)	0.063
Others	18/460	0/13	/	/	17/460	1/13	2.05 (0.25–16.63)	0.502	17/456	1/17	1.55 (0.19–12.36)	0.680
Clinical stages												
I + II + 4 s	160/460	13/13	**3.00 (1.35–6.64)**	**0.007**	163/460	10/13	2.20 (0.94–5.14)	0.068	156/456	17/17	**2.98 (1.48–6.01)**	**0.002**
III + IV	152/460	11/13	**2.69 (1.17–6.17)**	**0.019**	152/460	11/13	**2.64 (1.15–6.04)**	**0.022**	147/456	16/17	**3.03 (1.49–6.19)**	**0.002**

Abbreviations: CI, confidence interval; OR, odds ratio.

*Note*: Values are in bold if the *p* values are less than 0.05 or the 95% CIs excluded 1.

^a^ Adjusted for age and sex, omitting the correspondence factor.

### 3.3 Functional effect of rs3998860 G > A and rs12781492 A > C on surrounding genes

**FIGURE 1:**
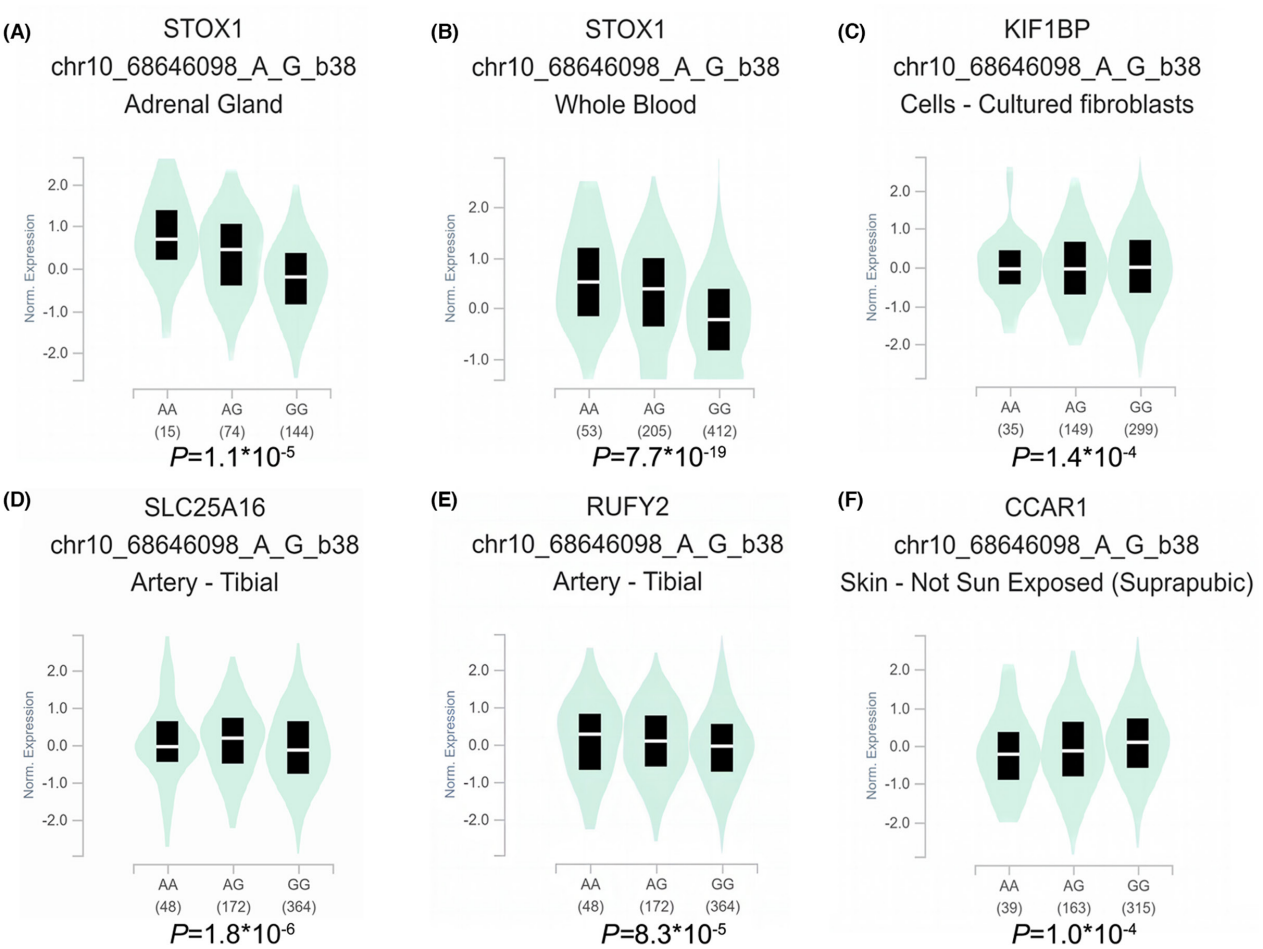
eQTL analysis shows the effect of *TET1* rs3998860 G > A on surrounding gene expression. (A, B) In the adrenal gland (*p* = 1.1 × 10^−5^) and whole blood (*p* = 7.7 × 10^−19^), rs3998860 G was significantly associated with decreased STOX1 mRNA expression. (C) In cultured fibroblasts, rs3998860 G was significantly associated with an increase in KIF1BP mRNA expression (*p* = 1.4 × 10^−4^). (D) In the tibial artery, rs3998860 A was significantly associated with increased mRNA expression of SLC25A16 (*p* = 1.8 × 10^−6^). (E) In the tibial artery, rs3998860 A was significantly associated with an increase in RUFY2 mRNA expression (*p* = 8.3 × 10^−5^). (F) In skin, rs3998860 G was significantly associated with increased mRNA expression of CCAR1 (*p* = 1.0 × 10^−4^). Violin diagrams were obtained through the GTEx official website (https://www.gtexportal.org/home/).

**FIGURE 2:**
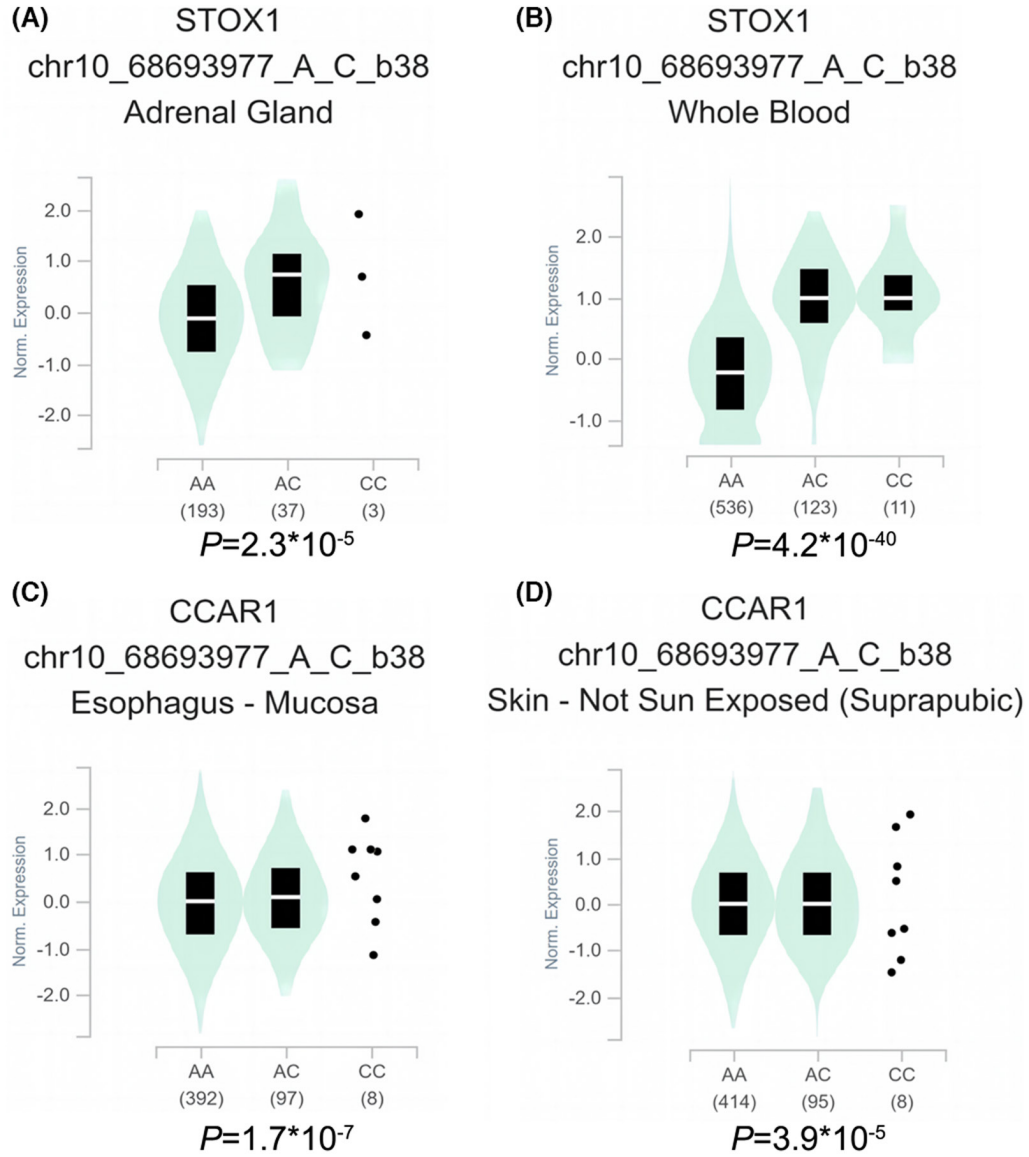
eQTL analysis shows the effect of *TET1* rs12781492 A > C on surrounding gene expression. (A, B) In the adrenal gland (*p* = 2.3 × 10^−5^) and whole blood (*p* = 4.2 × 10^−40^), rs12781492 C was significantly associated with increased STOX1 mRNA expression. (C, D) In the oesophageal mucosa (*p* = 1.7 × 10^−7^) and skin (*p* = 3.9 × 10^−5^), rs12781492 C was significantly associated with an increase in CCAR1 mRNA expression. Violin diagrams were obtained through the GTEx official website (https://www.gtexportal.org/home/).

Based on the impact of *TET1* rs3998860 G > A and rs12781492 A > C on susceptibility to neuroblastoma, we further explored the impact of these two loci on expression of nearby genes. We conducted cis‐eQTL analysis on rs3998860 (Figure 1) and rs12781492 (Figure 2) using the GTEx database. The results showed that the rs3998860 A allele was significantly associated with increased STOX1 mRNA expression in the adrenal gland (Figure 1A) and whole blood (Figure 1B) compared to the G allele. In cultured fibroblasts (Figure 1C), the A allele was significantly associated with increased KIF1BP mRNA expression compared to the rs3998860 G allele. In the tibial artery, the rs3998860 A allele was significantly associated with increased mRNA expression of SLC25A16 (Figure 1D) and RUFY2 (Figure 1E). In skin not exposed to sun, the rs3998860 A allele was significantly associated with decreased CCAR1 mRNA expression (Figure 1F). In the adrenal gland (Figure 2A) and whole blood (Figure 2B), the rs12781492 C allele was significantly associated with increased STOX1 mRNA expression compared to the rs12781492 A allele. The rs12781492 C allele significantly enhanced mRNA expression of CCAR1 in the oesophageal mucosa (Figure 2C) and skin not exposed to sun (Figure 2D) compared to the A allele.

### 3.4 Relationship between STOX1 mRNA expression and prognosis of neuroblastoma

**FIGURE 3:**
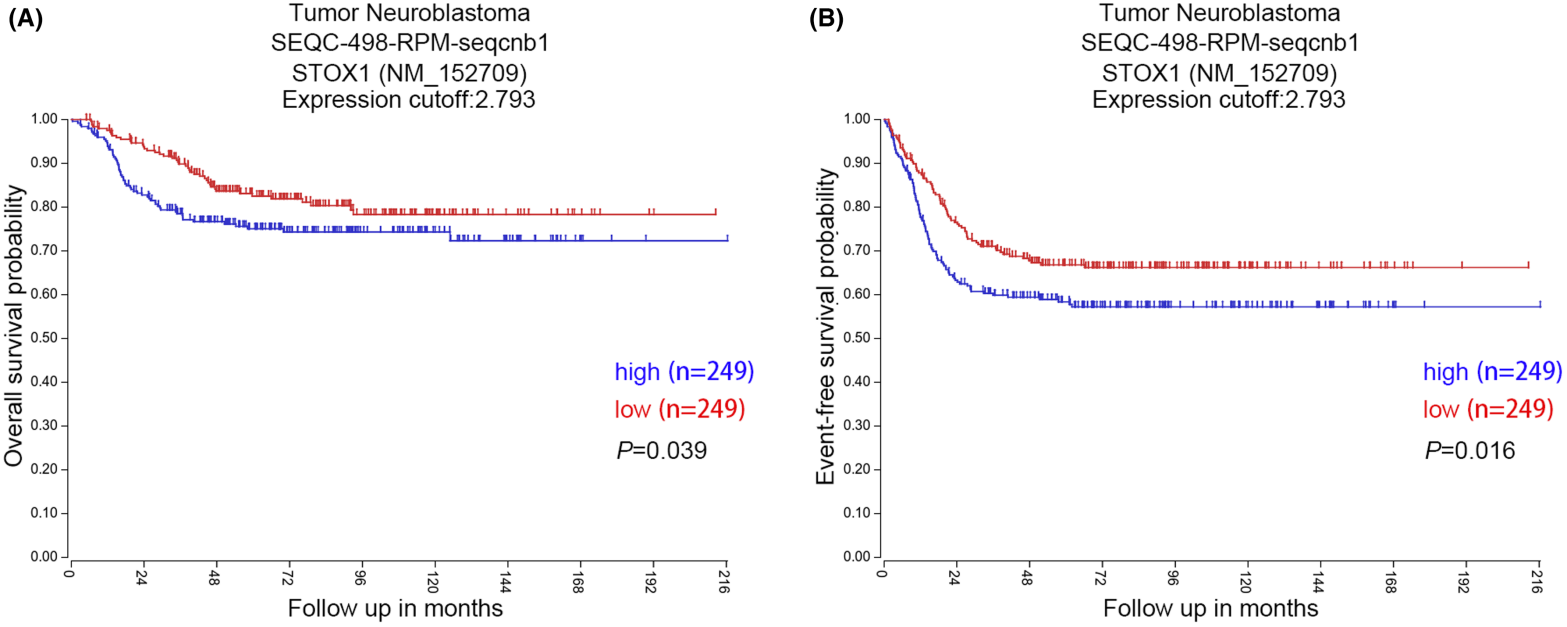
Kaplan–Meier analysis shows the relationship between STOX1 mRNA expression and prognosis. (A) In neuroblastomas, high mRNA expression of STOX1 was significantly associated with low OS (*p* = 0.039). (B) High mRNA expression of STOX1 was significantly associated with low EFS in neuroblastomas (*p* = 0.016).

Based on the significant impact of rs3998860 and rs12781492 on STOX1 mRNA expression in the adrenal gland and whole blood, we further analysed the relationship between mRNA expression of STOX1 and prognosis of neuroblastoma through the R2: Genomics Analysis and Visualization Platform (Figure 3). We conducted Kaplan–Meier survival analysis of overall survival (OS) and event‐free survival (EFS) prognostic indicators for neuroblastoma patients in the GSE62564 dataset. Neuroblastoma patients with high STOX1 (*n* = 249) mRNA expression had significantly (*p* = 0.039) lower OS than neuroblastoma patients with low STOX1 mRNA expression (*n* = 249; Figure 3A). Consistent with this, high mRNA expression of STOX1 was significantly (*p* = 0.016) associated with low EFS (Figure 3B).

## 4 DISCUSSION

Neuroblastoma is the most common tumour in children, and its high‐risk type has a high risk of recurrence even after multiple treatments.^3^, ^9^, ^10^, ^11^ Fundamentally understanding the genetic variations associated with neuroblastoma is key to early diagnosis and targeted treatment. Based on the important role of m5C modification in cancer, we focus on polymorphisms of m5C modification core genes. Our previous study showed that rs13181449 C > T in the m5C methyltransferase gene *NSUN2* confers reduced risk of neuroblastoma.^46^ In recent years, the m5C demethylase gene *TET1* has been proven to mediate the occurrence and development of some cancers.^41^, ^42^, ^43^, ^44^ However, the important role of *TET1* gene polymorphisms in cancer, including neuroblastoma, has not been revealed. Therefore, we explored the impact of *TET1* gene polymorphisms on risk of neuroblastoma through a case–control study. Our results suggest that *TET1* gene polymorphisms (rs3998860 G > A and rs12781492 A > C) are associated with increased neuroblastoma risk.

We used a case–control study to explore the association between risk of neuroblastoma and *TET1* gene polymorphisms in children in Jiangsu, China. Each SNP in our control group was found to conform to HWE, indicating that our data source is reliable. We conducted logistic regression analysis of the results of *TET1* gene polymorphism genotyping. The results showed that only the GG genotype of *TET1* rs3998860 significantly increased neuroblastoma risk compared to the AA genotype. Further model analysis showed significantly increased neuroblastoma risk only for the recessive model, consistent with the above results. In line with our expectations, the OR value in the recessive model also increased (OR: 2.69 > 2.51). Therefore, based on these results, the genetic model of rs3998860 in neuroblastoma is a recessive model, as is the genetic model of rs12781492 A > C in neuroblastoma. Combination of 1–3 risk genotypes significantly increased neuroblastoma risk when compared to the 0 risk genotype, with an OR value reaching the highest of all models. This indicates that risk SNPs can accumulate in neuroblastoma. Considering the effect of confounding factors, we further explored the association between *TET1* rs3998860 and susceptibility to neuroblastoma through stratification analysis. The results for the recessive model (AA vs. GG/GA) of *TET1* rs3998860 showed significantly increased neuroblastoma risk in subgroups >18 months of age and males. In site of origin stratification, recessive models of rs3998860 and rs12781492 indicated significantly increased risk of neuroblastoma only in the retroperitoneal subgroup, and the OR values were much higher in the other subgroups. This suggests that the variable site of origin might confound the results. rs3998860 G > A and rs12781492 A > C were only significantly associated with increased risk to retroperitoneal neuroblastoma, and the specific mechanisms need to be further studied. The recessive model of *TET1* rs3998860 showed significantly increased risk of neuroblastoma in all clinical staging subgroups. The recessive model (CC vs. AA/AC) of rs12781492 was significantly associated with neuroblastoma risk only in the retroperitoneal subgroup and the III + IV subgroup. In all subgroups of age, sex and clinical stage, as well as in the retroperitoneal subgroup, combination of 1–3 risk genotypes was significantly associated with increased neuroblastoma risk compared to the 0 risk genotype. Importantly, all models in the retroperitoneal subgroup were significant, indicating a significant association between *TET1* gene polymorphisms and increased risk to retroperitoneal neuroblastoma. These results indicate that there are indeed confounding factors interfering with our research conclusions, and our conclusions should thus be explained by a stratified subgroup.

To further explore the functions of rs3998860 and rs12781492, we used the GTEx database to analyse their impact on nearby gene expression. Importantly, we found that both the rs3998860 A allele and the rs12781492 C allele were significantly associated with increased STOX1 mRNA expression in the adrenal gland and whole blood. Kaplan–Meier analysis showed that high mRNA expression of STOX1 was associated with poor prognosis. Interestingly, our findings precisely indicate that the rs3998860 A allele and the rs12781492 C allele are associated with increased neuroblastoma risk. The *STOX1* (storkhead Box 1) gene has been implicated in pre‐eclampsia.^47^ Expression of the largest isoform of STOX1 (STOX1A) activates the PI3K‐Akt‐FOX pathway in the nucleus and inhibits the pathway in the cytoplasm.^47^ Gao et al. demonstrated that STOX1 can inhibit medulloblastoma by inhibiting Math1 expression and that activation of sonic hedgehog signalling can inhibit STOX1 and restore Math1 expression.^48^ Importantly, STOX1A has been shown to directly regulate expression of mitotic cyclin CCNB1 in the SH‐SY5Y neuroblastoma cell line and participate in regulating the cell cycle.^49^ Consistent with our findings, knockdown of STOX1A has been shown to inhibit proliferation by neuroblastoma cells.^49^ Our study reveals the unique cancer‐promoting effect of STOX1 in neuroblastoma compared to other tumours. In other words, rs3998860 and rs12781492 may affect neuroblastoma susceptibility and prognosis by influencing STOX1 mRNA expression. In skin not exposed to sun, both the rs3998860 G allele and the rs12781492 C allele significantly enhanced mRNA expression of CCAR1. The apoptosis regulatory factor CCAR1 plays an important role in promoting cancer,^50^, ^51^ and deletion of CCAR1 inhibits the occurrence and development of prostate cancer cells.^50^ CCAR1 is an important activator for maintaining the growth of breast cancer.^51^ Consistent with our findings, high expression of CCAR1 is associated with cancer occurrence and development. *TET1* rs3998860 and rs12781492 may increase neuroblastoma risk by influencing mRNA expression of CCAR1. In cultured fibroblasts, the rs3998860 A allele was significantly associated with increased KIF1BP mRNA expression compared to the G allele. The relationship between the protein family member 1 binding protein KIF1BP and tumours is unclear. Studies have shown that KIF1BP is necessary for the growth and maintenance of nerve axons.^52^ In general, neuroblastomas with a lower degree of differentiation are less malignant.^53^ This is inconsistent with our research results. The role of KIF1BP in neuroblastoma needs to be further elucidated. In the tibial artery, the rs3998860 A allele was significantly associated with increased mRNA expression of SLC25A16 and RUFY2. There is a lack of research on SLC25A16. The RUN and FYVE domain containing 2 (RUFY2) gene has been found to frequently be mutated in high‐microsatellite instability cancers.^54^ RUFY2 expression correlates negatively with risk of glioblastoma.^55^ There are almost no relevant studies on the *RUFY2* gene in neuroblastoma, and the relationship between the *RUFY2* gene and neuroblastoma remains to be elucidated. The GTEx database did not reveal a relationship between expression of the *TET1* gene and rs3998860 and rs12781492. Further research is needed to clarify this in the future.

Our study is the first to clarify the association between *TET1* gene polymorphisms and susceptibility to neuroblastoma in children in Jiangsu, China, and its possible mechanism. Our sample size was relatively large, and comparability between the case group and the control group was high. Nevertheless, there are shortcomings in this study. First, the subjects included were only recruited from one hospital in Nanjing, China, limiting the extension of the conclusion. Second, the predicted functional SNPs examined may be biased, and functional SNPs that affect neuroblastoma may be missed. Finally, the specific mechanism of *TET1* rs3998860 and rs12781492 in neuroblastoma remains to be elucidated.

In summary, our study demonstrates that *TET1* gene rs3998860 G > A and rs12781492 A > C significantly increase neuroblastoma risk. The potential mechanisms of *TET1* gene polymorphisms in neuroblastoma need to be further elucidated.

## AUTHOR CONTRIBUTIONS

**Jiaming Chang:** Investigation (equal); writing – original draft (equal); writing – review and editing (equal). **Lei Lin:** Formal analysis (equal); investigation (equal); methodology (equal); writing – original draft (equal); writing – review and editing (equal). **Chunlei Zhou:** Data curation (equal); investigation (equal); resources (equal); writing – review and editing (equal). **Xinxin Zhang:** Investigation (equal); writing – review and editing (equal). **Tianyou Yang:** Investigation (equal); writing – review and editing (equal). **Haiyan Wu:** Data curation (equal); investigation (equal); resources (equal); writing – review and editing (equal). **Yan Zou:** Conceptualization (equal); investigation (equal); supervision (equal); writing – review and editing (equal). **Jing He:** Conceptualization (equal); formal analysis (equal); funding acquisition (equal); investigation (equal); methodology (equal); supervision (equal); writing – review and editing (equal).

## CONFLICT OF INTEREST STATEMENT

None declared.

## Supporting information


Table S1.

## Data Availability

All the data are available upon request from the corresponding authors.
